# Pharmacovigilance study of anti-infective-related acute kidney injury using the Japanese adverse drug event report database

**DOI:** 10.1186/s40360-021-00513-x

**Published:** 2021-08-30

**Authors:** Satoshi Nakao, Shiori Hasegawa, Ryogo Umetsu, Kazuyo Shimada, Ririka Mukai, Mizuki Tanaka, Kiyoka Matsumoto, Yu Yoshida, Misaki Inoue, Riko Satake, Yuri Nishibata, Jun Liao, Mitsuhiro Nakamura

**Affiliations:** 1 Laboratory of Drug Informatics, Gifu Pharmaceutical University; Gifu, 1-25-4, Daigaku-Nishi, Gifu, 501-1196 Japan; 2 Present Address: Department of Pharmacy, Kyushu University Hospital, Fukuoka, Japan; 3 Division of Pharmacy, Kobe City Medical Center General Hospital, Kobe, Hyogo Japan; 4 Present Address: Micron Inc, Tokyo, Japan; 5 Present Address: Division of Pharmacy, Japanese Red Cross Wakayama Medical Center, Wakayama, Japan; 6 Department of Pharmaceutical Informatics and Biological Statistics, School of Science, China Pharmaceutical University, Nanjing, China

**Keywords:** Acute kidney injury, Anti-infective, Japanese adverse drug event report database, Propensity score, JADER, Polypharmacy

## Abstract

**Background:**

Acute kidney injury (AKI) is associated with significant increases in short- and long-term morbidity and mortality. Drug-induced AKI is a major concern in the present healthcare system. Our spontaneous reporting system (SRS) analysis assessed links between AKI, along with patients’ age, as healthcare-associated risks and administered anti-infectives. We also generated anti-infective-related AKI-onset profiles.

**Method:**

We calculated reporting odds ratios (RORs) for reports of anti-infective-related AKI (per Medical Dictionary for Regulatory Activities) in the Japanese Adverse Drug Event Report database and evaluated the effect of anti-infective combination therapy. The background factors of cases with anti-infective monotherapy and combination therapy (≥ 2 anti-infectives) were matched using propensity score. We evaluated time-to-onset data and hazard types using the Weibull parameter.

**Results:**

Among 534,688 reports (submission period: April 2004–June 2018), there were 21,727 AKI events. The reported number of AKI associated with glycopeptide antibacterials, fluoroquinolones, third-generation cephalosporins, triazole derivatives, and carbapenems were 596, 494, 341, 315, and 313, respectively. Crude RORs of anti-infective-related AKI increased among older patients and were higher in anti-infective combination therapies [anti-infectives, ≥ 2; ROR, 1.94 (1.80–2.09)] than in monotherapies [ROR, 1.29 (1.22–1.36)]. After propensity score matching, the adjusted RORs of anti-infective monotherapy and combination therapy (≥ 2 anti-infectives) were 0.67 (0.58–0.77) and 1.49 (1.29–1.71), respectively. Moreover, 48.1% of AKI occurred within 5 days (median, 5.0 days) of anti-infective therapy initiation.

**Conclusion:**

RORs derived from our new SRS analysis indicate potential AKI risks and number of administered anti-infectives.

**Supplementary Information:**

The online version contains supplementary material available at 10.1186/s40360-021-00513-x.

## Background

Acute kidney injury (AKI) is associated with significant increase in short- and long-term morbidity and mortality [1] and occurs in approximately 1–5% of all patients treated at the hospital [2]. AKI is a sudden episode of kidney failure or kidney damage that happens within a few hours or a few days [1, 3]. Drug-induced AKI has been implicated in 8 to 60% of all cases of in-hospital AKI and as such is a recognized source of significant morbidity and mortality [4]. Healthcare professionals should be aware of potential AKI risk since occasional fatalities have also been observed. Thus, drug-induced AKI is a major concern in the present healthcare system. AKI is pre-renally caused by cardiovascular disorders and hypovolemia, intra-renally caused by acute tubular necrosis and other parenchymal disorders, or post-renally caused by bladder obstruction and ureteral obstruction [2, 4, 5]. Several antibiotics including penicillin analogs, cephalosporins, and ciprofloxacin are known to increase the risk of intra-renal AKI [5], and other antibiotics such as aminoglycosides, amphotericin B, and vancomycin have been identified as the cause of adverse events (AEs) in AKI [4].

Spontaneous reporting systems (SRSs), such as the US Food and Drug Administration (FDA) adverse event reporting system (FAERS) and the Japanese Adverse Drug Event Report (JADER) database, have been used in pharmacovigilance assessments [6–12]. The reporting odds ratio (ROR) has been used to derive an index for detecting drug-associated adverse events (AEs) [13, 14]. A previous study using the FAERS demonstrated that vancomycin, trimethoprim–sulfamethoxazole, piperacillin–tazobactam, and ciprofloxacin have significant reporting associations with AKI [6]; however, the study did not include less frequently used colistin and aminoglycosides and their association with AKI. Patek et al. conducted a more detailed study focused on anti-infectives using the FAERS database [7]. It has been reported that piperacillin-tazobactam and vancomycin were frequently reported to be associated with AKI using the JADER database [8]. However, the detailed time-to-onset profiles of antibiotics was not established clearly in SRSs; therefore, we focused on this aspect in our present study.

Polypharmacy is a well-known risk factor for AEs. Altered liver and kidney functions are considered a cause for changes in the pharmacokinetics in elderly patients [15]. Older patients often suffer from multiple diseases and receive several drugs, which is referred to as polypharmacy [16–18]. Pierson-Marchandise et al. suggested that AKI risk is particularly high corresponding to polypharmacy, and increases proportionate to the number of drugs administered [19]. We previously analyzed an SRS database and found that the combination of medications might increase the risk of AEs according to the index derived from the RORs [10, 11]. Using a multivariate logistic regression analysis technique, Abe et al. showed that the number of drugs administered and age might be more closely linked to an increased risk of kidney disorder than liver disorder [10]. Recently, propensity score (PS) matching has been used as an assessment approach to reduce selection bias by equating groups based on covariates or other appropriate parameters [20]. Its usefulness has been evident from the analysis of AE reports in the FAERS database [9]. In this study, we evaluated the anti-infective-related AKI profiles using ROR, the time-to-onset data of AKI with respect to anti-infective therapy initiation, and the effect of the number of anti-infectives administered in a clinical setting using real-world data.

## Methods

The JADER dataset is publicly available and can be downloaded from the website of the Pharmaceuticals and Medical Devices Agency (PMDA) (www.pmda.go.jp). This study used a dataset containing information recorded between April 2004 and June 2018. The JADER database consists of four tables: patients’ demographic information (DEMO), drug information (DRUG), AE information (REAC), and primary disease information (HIST). The four data tables imported to the relational database (FileMaker Pro 14 software (FileMaker, Santa Clara, CA, USA)). The “DRUG” table contains the role code assigned to each drug: “suspected drug,” “concomitant drug,” and “interacting drug.” All drugs in the “suspected drug,” “concomitant drug,” and “interacting drug” association classes were used for the analyses.

The AEs in the JADER database are coded according to the preferred terminology by the Medical Dictionary for Regulatory Activities/Japanese (MedDRA/J) version 19.0 (MedDRA/J, www.pmrj.jp/jmo/php/indexj.php). The MedDRA dictionary is organized with a five-level hierarchy, including System Organ Class (SOC), High-Level Group Terms (HLGT), High-Level Terms (HLT), Preferred Terms (PT), and Lowest Level Terms (LLT). Preferred Terms (PTs) represent more precise medical terminology. Several studies on AKI have been reported; however, we could not find a standard criterion for the selection of PTs in each category. Patek et al. and Hosohata et al. selected a PT of “acute kidney injury” in a study on AKI [7, 8]. Welch et al. used 22 search terms based on AKI expert opinion [6]. Standardized MedDRA Queries (SMQ) are groupings of MedDRA terms, ordinarily PTs, that relate to a defined medical condition [21]. Pierson-Marchandise et al. selected 44 PTs according to SMQ (code: 20000003) [19]. Selection of a large number of PTs such as SMQ generally allows identification of all possible cases inclusive of less-specific cases. Selection of small number of PTs allows identification of cases that precisely define the condition of interest. We selected 19 PTs to extract case reports of AKI-related AEs based on SMQ and previous reports (Table 1). To identify an AE signal, we calculated crude ROR by using a two-by-two contingency table [14, 22]. The RORs indicated the presence or absence of a particular drug and a particular AE in the database, and were expressed as point estimates with 95% confidence intervals (CIs). The signal of a drug-AE combination was considered statistically significant when the estimated ROR and lower limit of the corresponding 95% CI was greater than one. The positive identification of a signal required two or more cases [14, 22].

**Table 1:** List of the preferred terms of acute kidney injury-related adverse events

PT^a^	PT^a^ code
Acute kidney injury	10069339^b^
Albuminuria	10001580^b^
Azotaemia	10003885^b^
Blood creatinine abnormal	10005481^b^
Blood creatinine increased	10005483^b^
Blood urea abnormal	10005846^b^
Blood urea increased	10005851^b^
Creatinine renal clearance abnormal	10068447^b^
Creatinine renal clearance decreased	10011372^b^
Glomerular filtration rate decreased	10018358^b^
Hypercreatininaemia	10062747^b^
Oedema due to renal disease	10049630^b^
Protein urine present	10053123^b^
Proteinuria	10037032^b^
Renal disorder	10038428
Renal failure	10038435^b^
Renal function test abnormal	10061480^b^
Renal impairment	10062237^b^
Renal tubular disorder	10038537^b^

^a^ Preferred Term

^b^ PTs included in the standardized MedDRA query ‘acute renal failure’

The propensity score (PS) matching is a statistical matching technique to construct matched sets with similar distributions of the covariates, without requiring close or exact matches on all of the individual variables [20]. The following variables were included in the multiple logistic regression model: age, body weight, height, sepsis, and number of anti-infectives administered. The presence or absence of AKI was evaluated as the outcome. Patients with sepsis related terms input to “REAC” and “HIST” tables were considered to have a sepsis background (Table 2). Data was not arranged according to the severity of the disease because it was not included in the case reports extracted from the JADER database. Nearest neighbor matching was performed based on the calculated PS between anti-infective monotherapy and combination therapy (≥ 2 anti-infectives). A caliper width of 0.2 of the standard deviation of the logit of PS was used. The standard mean difference (SMD) was used as a covariate balance indicator between anti-infective monotherapy and combination therapy. The SMD values below 0.1 were considered optimal for an adequate covariate balance.

**Table 2:** List of the preferred terms of Sepsis-related adverse events

PT^a^	PT^a^ code
Bacterialsepsis	10053840
Corynebacteriumsepsis	10057767
Devicerelatedsepsis	10069802
Enterobactersepsis	10054219
Enterococcalsepsis	10054221
Escherichiasepsis	10015296
GroupBstreptococcusneonatalsepsis	10053588
Haemophilussepsis	10058875
Helicobactersepsis	10054264
Klebsiellasepsis	10054160
Listeriasepsis	10063085
Neutropenicsepsis	10049151
Pneumococcalsepsis	10054047
Pseudomonalsepsis	10058877
Sepsis	10040047
Sepsisneonatal	10040049
Septicembolus	10040067
Septicshock	10040070
Staphylococcalsepsis	10056430
Stenotrophomonassepsis	10054137
Streptococcalsepsis	10048960
Urosepsis	10048709

^a^ Preferred Term

To assess the time-to-onset profile, the median time from the first prescription of each report to the onset of AKI was used in conjunction with the interquartile range and Weibull shape parameter (WSP) [12, 23]. We selected an analysis period of 90 days after therapy initiation. The rate of occurrence of AEs after prescription is thought to depend on the causal mechanism. The WSP represents the failure rate distribution against time. A larger scale value (α) of the Weibull distribution indicates a wider data distribution. A smaller scale value (α) shrinks the data distribution. The WSP (β) has been used to determine the level of hazard over time without a reference population. When β is equal to 1, the hazard is considered to be constant over time. When β was lower than 1, the hazard was considered to decrease over time (initial failure type). In contrast, when β was greater than 1, the hazard was considered to increase over time (wear-out failure type) [23]. The results obtained from the WSP are complementary to the results of the disproportionality analysis using ROR.

These data analyses were performed using JMP Pro 16.0 (SAS Institute, Cary, NC, USA).

## Results

The JADER database contains 534,688 reports submitted between April 2004 and June 2018, and we identified 21,727 AKI events. According to the Anatomical Therapeutic Chemical (ATC) Classification System (www.whocc.no/atc_ddd_index/), 145 anti-infectives were selected and categorized into 36 ATC-drug classes (S1 Table).

In the top five anti-infective therapies, glycopeptide antibacterials (ATC code: J01XA), fluoroquinolones (ATC code: J01MA), third-generation cephalosporins (ATC code: J01DD), triazole derivatives (ATC code: J02AC), and carbapenems (ATC code: J01DH), we identified 596, 494, 341, 315, and 313 reported AKI-associated AEs, respectively (Table 3). The lower limit of the 95% CI (confidence interval) of ROR was > 1 for the following drug groups: combinations of penicillins, including beta-lactamase inhibitors (ATC code: J01CR), second-generation cephalosporins (ATC code: J01DC), third-generation cephalosporins (ATC code: J01DD), fourth-generation cephalosporins (ATC code: J01DE), monobactams (ATC code: J01DF), carbapenems (ATC code: J01DH), intermediate-acting sulfonamides (ATC code: J01EC), combinations of sulfonamides and trimethoprim, including derivatives (ATC code: J01EE), lincosamides (ATC code: J01FF), other aminoglycosides (ATC code: J01GB), fluoroquinolones (ATC code: J01MA), other quinolones (ATC code: J01MB), glycopeptide antibacterials (ATC code: J01XA), polymyxins (ATC code: J01XB), other antibacterials (ATC code: J01XX), antibiotics (ATC code: J02AA), triazole derivatives (ATC code: J02AC), and other antimycotics for systemic use (ATC code: J02AX).

**Table 3:** Number of reports and crude reporting odds ratio of acute kidney injury associated with anti-infectives

Classification (ATC code)	Total (n)	Case (n)	Crude ROR^a^ (95% CI^b^)
Total	534,688	21,727	
Antibacterials			
Tetracyclines (J01AA)	1763	75	1.05 (0.83–1.32)
Amphenicols (J01BA)	49	0	–^c^
Penicillins with extended spectrum (J01CA)	3264	121	0.91 (0.76–1.09)
Beta-lactamase sensitive penicillins (J01CE)	93	6	1.63 (0.71–3.72)
Beta-lactamase resistant penicillins (J01CF)	0	0	–^c^
Beta-lactamase inhibitors (J01CG)	0	0	–^c^
Combinations of penicillins, incl. Beta-lactamase inhibitors (J01CR)	2121	292	3.81 (3.36–4.31)^d^
First-generation cephalosporins (J01DB)	1484	46	0.75 (0.56–1.01)
Second-generation cephalosporins (J01DC)	1902	101	1.33 (1.08–1.62)^d^
Third-generation cephalosporins (J01DD)	7551	341	1.12 (1.002–1.25)^d^
Fourth-generation cephalosporins (J01DE)	1318	128	2.55 (2.12–3.06)^d^
Monobactams (J01DF)	21	4	5.56 (1.87–16.51)^d^
Carbapenems (J01DH)	3551	313	2.30 (2.05–2.59)^d^
Other cephalosporins and penems (J01DI)	132	9	1.73 (0.88–3.40)
Trimethoprim and derivatives (J01EA)	0	0	–^c^
Intermediate-acting sulfonamides (J01EC)	36	7	5.70 (2.50–13.01)^d^
Long-acting sulfonamides (J01ED)	4	0	–^c^
Combinations of sulfonamides and trimethoprim, incl. Derivatives (J01EE)	2738	261	2.51 (2.20–2.85)^d^
Macrolides (J01FA)	5283	200	0.93 (0.81–1.07)
Lincosamides (J01FF)	757	55	1.85 (1.41–2.44)^d^
Streptogramins (J01FG)	2	0	–^c^
Streptomycins (J01GA)	153	9	1.48 (0.75–2.89)
Other aminoglycosides (J01GB)	932	258	9.13 (7.91–10.55)^d^
Fluoroquinolones (J01MA)	8571	494	1.45 (1.33–1.59)^d^
Other quinolones (J01MB)	24	3	3.37 (1.01–11.31)^d^
Combinations of antibacterials (J01RA)	638	11	0.41 (0.23–0.75)
Glycopeptide antibacterials (J01XA)	2752	596	6.68 (6.10–7.32)^d^
Polymyxins (J01XB)	137	107	84.62 (56.43–126.89)^d^
Steroid antibacterials (J01XC)	2	0	–^c^
Imidazole derivatives (J01XD)	667	12	0.43 (0.24–0.77)
Nitrofuran derivatives (J01XE)	0	0	–^c^
Other antibacterials (J01XX)	2119	107	1.26 (1.03–1.53)^d^
Antimycotics			
Antibiotics (J02AA)	1337	299	6.88 (6.05–7.83)^d^
Imidazole derivatives (J02AB)	100	1	–^c^
Triazole derivatives (J02AC)	3130	315	2.67 (2.37–3.00)^d^
Other antimycotics for systemic use (J02AX)	1148	161	3.87 (3.28–4.58)^d^

^a^ Reporting Odds Ratio, ^b^ Confidence Interval, ^c^ Number of cases < 2, ^d^ Lower limit of 95% CI > 1

AKI crude RORs (95% CI) for vancomycin, tazobactam/piperacillin, and vancomycin plus tazobactam/piperacillin were 4.00 (3.69–4.33), 3.48 (3.16–3.83), and 6.07 (4.96–7.43), respectively (Table 4). The crude RORs (95% CI) of age (80–89, and ≥ 90 years), and the number of anti-infectives administered (1 and ≥ 2) were 1.58 (1.52–1.64), 1.89 (1.74–2.06), 1.29 (1.22–1.36), and 1.94 (1.80–2.09), respectively (Table 5). The Receiver Operating Characteristic (ROC) curve of the PS determine the accuracy of the model predictions of treatment allocation. The area under the ROC curve was 0.5766 (data not shown). Since the SMD of each factor was below 0.1, the background factors of cases with anti-infective monotherapy and ≥ 2 anti-infectives were matched (Table 6). After the PS matching, the adjusted RORs of anti-infective monotherapy and combination therapy (≥ 2 anti-infectives) were 0.67 (0.58–0.77) and 1.49 (1.29–1.71), respectively.

**Table 4:** Crude reporting odds ratio of acute kidney injury with vancomycin combination

Antibiotics or Antibiotic Combinations	Total (n)	Case (n)	Crude ROR^a^ (95% CI^b^)
Vancomycin	5101	723	4.00 (3.69–4.33)
Sulfamethoxazole-Trimethoprim	15,548	938	1.54 (1.44–1.65)
Vancomycin plus Sulfamethoxazole-Trimethoprim	702	76	2.87 (2.26–3.65)
Levofloxacin	11,764	627	1.34 (1.23–1.45)
Vancomycin plus Levofloxacin	563	63	2.98 (2.29–3.87)
Clarithromycin	8040	318	0.97 (0.87–1.09)
Vancomycin plus Clarithromycin	99	11	2.95 (1.58–5.53)
Meropenem	7303	611	2.19 (2.01–2.38)
Vancomycin plus Meropenem	1678	184	2.92 (2.51–3.41)
Fluconazole	4961	398	2.08 (1.87–2.31)
Vancomycin plus Fluconazole	430	53	3.32 (2.49–4.43)
Ceftriaxone	4772	296	1.57 (1.39–1.77)
Vancomycin plus Ceftriaxone	351	44	3.39 (2.47–4.65)
Itraconazole	4354	254	1.47 (1.29–1.67)
Vancomycin plus Itraconazole	305	41	3.67 (2.64–5.10)
Cefazolin	4266	198	1.15 (0.997–1.33)
Vancomycin plus Cefazolin	298	21	1.79 (1.15–2.79)
Cefcapene pivoxil	4262	182	1.05 (0.91–1.22)
Vancomycin plus Cefcapene pivoxil	58	5	2.23 (0.89–5.57)
Minocycline	4066	218	1.34 (1.17–1.54)
Vancomycin plus Minocycline	350	43	3.31 (2.41–4.56)
Micafungin	4008	437	2.93 (2.65–3.24)
Vancomycin plus Micafungin	905	125	3.80 (3.14–4.59)
Cefepime	3971	299	1.94 (1.72–2.18)
Vancomycin plus Cefepime	652	88	3.69 (2.95–4.63)
Tazobactam/piperacillin	3812	483	3.48 (3.16–3.83)
Vancomycin plus Tazobactam/piperacillin	584	119	6.07 (4.96–7.43)

^a^ Reporting Odds Ratio, ^b^ Confidence Interval

**Table 5:** Crude reporting odds ratio of acute kidney injury

	Total	Case^a^	Crude ROR^b^ (95% CI^c^)
Total	534,688	21,727	
Reporting year			
Male	260,713	11,988	1.31 (1.27–1.34)
Age			
≤ 19 years	37,941	992	0.62 (0.58–0.66)
20–29 years	17,049	440	0.62 (0.56–0.68)
30–39 years	29,004	704	0.57 (0.53–0.62)
40–49 years	38,486	1247	0.78 (0.73–0.82)
50–59 years	63,734	2266	0.86 (0.82–0.89)
60–69 years	113,007	4603	1.00 (0.97–1.04)
70–79 years	122,114	5527	1.16 (1.12–1.20)
80–89 years	61,022	3606	1.58 (1.52–1.64)
≥ 90 years	8202	602	1.89 (1.74–2.06)
Anti-infectives (Antibacterials+Antimycotics)			
0 drug	495,039	19,472	0.68 (0.65–0.71)
1 drug	29,938	1529	1.29 (1.22–1.36)
≥ 2 drugs	9711	726	1.94 (1.80–2.09)

^a^ Number of patients with acute kidney injury, ^b^ Reporting Odds Ratio, ^c^ Confidence Interval

**Table 6:** Comparison of the number of reports for each factor before and after propensity score matching

	before propensity score matching				after propensity score matching			
	≥ 2 drugs (n = 9,711)	1 drug (n = 29,938)	p	Standard mean difference	≥ 2 drugs (n = 6,044)	1 drug (n = 6,044)	p	Standard mean difference
Sex			< 0.0001^*^	0.0985			0.5788	0.0100
Male	5683 (0.5861)	16037 (0.5373)			3561 (0.5892)	3591 (0.5941)		
Female	4014 (0.4139)	13809 (0.4627)			2483 (0.4108)	2453 (0.4059)		
Age (year)			< 0.0001^*^				0.9991	
< 10	669 (0.0693)	1671 (0.0563)			358 (0.0592)	363 (0.0601)		
10	462 (0.0479)	1183 (0.0399)			285 (0.0472)	265 (0.0438)		
20	477 (0.0494)	1586 (0.0534)			238 (0.0394)	225 (0.0372)		
30	599 (0.0621)	2367 (0.0797)			344 (0.0569)	341 (0.0564)		
40	723 (0.0749)	2186 (0.0736)			455 (0.0753)	448 (0.0741)		
50	1256 (0.1302)	3506 (0.1181)			812 (0.1343)	819 (0.1355)		
60	2095 (0.2171)	5787 (0.1949)			1382 (0.2287)	1399 (0.2315)		
70	2195 (0.2275)	6903 (0.2325)			1476 (0.2442)	1485 (0.2457)		
80	1047 (0.1085)	3821 (0.1287)			636 (0.1052)	643 (0.1064)		
90	122 (0.0126)	669 (0.0225)			57 (0.0094)	55 (0.0091)		
100	3 (0.0003)	7 (0.0002)			1 (0.0002)	1 (0.0002)		
Height (cm)			< 0.0001^*^				0.9998	
< 10	0 (0.0000)	0 (0.0000)			0 (0.0000)	0 (0.0000)		
10	0 (0.0000)	1 (0.0001)			0 (0.0000)	0 (0.0000)		
20	3 (0.0005)	1 (0.0001)			2 (0.0003)	1 (0.0002)		
30	31 (0.0050)	19 (0.0010)			15 (0.0025)	15 (0.0025)		
40	22 (0.0035)	44 (0.0024)			20 (0.0033)	21 (0.0035)		
50	21 (0.0034)	44 (0.0024)			20 (0.0033)	26 (0.0043)		
60	29 (0.0047)	62 (0.0033)			28 (0.0046)	27 (0.0045)		
70	52 (0.0084)	111 (0.0060)			48 (0.0079)	47 (0.0078)		
80	46 (0.0074)	85 (0.0046)			43 (0.0071)	45 (0.0074)		
90	53 (0.0085)	88 (0.0047)			49 (0.0081)	51 (0.0084)		
100	42 (0.0068)	98 (0.0053)			40 (0.0066)	41 (0.0068)		
110	42 (0.0068)	114 (0.0061)			42 (0.0069)	39 (0.0065)		
120	58 (0.0093)	132 (0.0071)			58 (0.0096)	62 (0.0103)		
130	102 (0.0164)	403 (0.0217)			99 (0.0164)	88 (0.0146)		
140	747 (0.1202)	2449 (0.1319)			727 (0.1203)	723 (0.1196)		
150	1889 (0.3038)	5975 (0.3218)			1847 (0.3056)	1824 (0.3018)		
160	2108 (0.3391)	6222 (0.3351)			2058 (0.3405)	2084 (0.3448)		
170	890 (0.1432)	2477 (0.1334)			868 (0.1436)	871 (0.1441)		
180	81 (0.0130)	238 (0.0128)			79 (0.0131)	79 (0.0131)		
190	1 (0.0002)	4 (0.0002)			1 (0.0002)	0 (0.0000)		
≥ 200	0 (0.0000)	0 (0.0000)			0 (0.0000)	0 (0.0000)		
Weight (kg)			0.0003^*^				0.9916	
< 10	200 (0.0264)	369 (0.0177)			117 (0.0194)	120 (0.0199)		
10	264 (0.0348)	620 (0.0298)			162 (0.0268)	165 (0.0273)		
20	190 (0.0250)	470 (0.0226)			119 (0.0197)	113 (0.0187)		
30	597 (0.0787)	1683 (0.0808)			437 (0.0723)	438 (0.0725)		
40	1813 (0.2390)	4929 (0.2366)			1498 (0.2478)	1492 (0.2469)		
50	2213 (0.2917)	6176 (0.2964)			1844 (0.3051)	1851 (0.3063)		
60	1506 (0.1985)	4135 (0.1985)			1204 (0.1992)	1212 (0.2005)		
70	559 (0.0737)	1672 (0.0802)			457 (0.0756)	465 (0.0769)		
80	156 (0.0206)	503 (0.0241)			132 (0.0218)	131 (0.0217)		
90	47 (0.0062)	157 (0.0075)			44 (0.0073)	32 (0.0053)		
100	15 (0.0020)	62 (0.0030)			9 (0.0015)	10 (0.0017)		
110	12 (0.0016)	19 (0.0009)			10 (0.0017)	6 (0.0010)		
120	4 (0.0005)	7 (0.0003)			1 (0.0002)	0 (0.0000)		
130	0 (0.0000)	3 (0.0001)			0 (0.0000)	0 (0.0000)		
140	4 (0.0005)	3 (0.0001)			3 (0.0005)	2 (0.0003)		
150	3 (0.0004)	11 (0.0005)			3 (0.0005)	3 (0.0005)		
160	3 (0.0004)	7 (0.0003)			3 (0.0005)	4 (0.0007)		
170	0 (0.0000)	5 (0.0002)			0 (0.0000)	0 (0.0000)		
180	0 (0.0000)	2 (0.0001)			0 (0.0000)	0 (0.0000)		
190	0 (0.0000)	0 (0.0000)			0 (0.0000)	0 (0.0000)		
≥ 200	1 (0.0001)	2 (0.0001)			1 (0.0002)	0 (0.0000)		
Sepsis			< 0.0001^*^	0.2190			0.7867	0.0048
1	799 (0.0823)	953 (0.0318)			478 (0.0791)	470 (0.0778)		
0	8912 (0.9177)	28985 (0.9682)			5566 (0.9209)	5574 (0.9222)		

* P< 0.05

Combinations containing the complete information on the treatment start date and AE onset date were extracted for the time-to-onset analysis. We evaluated 14 anti-infective categories for which the number of cases was more than 100 and the lower limit of the 95% CI exceeded 1 as shown in Table 3 (Table 7, S1 Figure). S1 Figure shows a histogram of the number of AKI onsets in relation to the number of days after anti-infective treatment initiation (from day 0 to day 90). The median period (interquartile range) until AKI onset caused by anti-infectives was 5.0 (2.0–11.0) days for orally (per os, po) administered anti-infectives and 5.0 (2.0–9.0) days for administration by intravenous (iv) injection. The upper limits of the 95% CI of the β value were less than 1 for po administered anti-infectives.

**Table 7:** The medians and Weibull parameter of each drug

Classification (ATC code)	Administration route	Case	Median (day)	Scale parameter	Shape parameter
	(po: per os; iv: intravenous injection)	(n)	(25–75%)	α (95% CI^a^)	β (95% CI^a^)
Total	po	756	5.0 (2.0–11.0)	10.60 (9.68–11.59)	0.87 (0.82–0.92)
	iv	1800	5.0 (2.0–9.0)	8.28 (7.88–8.71)	1.03 (0.99–1.06)
Antibacterials					
Combinations of penicillins, incl. Beta-lactamase inhibitors (J01CR)	po	9	3.0 (2.5–11.5)	8.00 (1.94–30.36)	0.65 (0.34–1.06)
	iv	180	4.0 (2.0–8.0)	7.43 (6.34–8.69)	1.04 (0.93–1.15)
Second-generation cephalosporins (J01DC)	po	8	4.5 (1.5–9.0)	6.95 (3.85–12.12)	1.79 (0.83–3.20)
	iv	56	3.0 (1.0–5.8)	5.16 (3.86–6.82)	1.06 (0.85–1.30)
Third-generation cephalosporins (J01DD)	po	93	3.0 (1.0–7.0)	7.06 (5.47–9.06)	0.90 (0.77–1.05)
	iv	138	4.0 (2.0–7.0)	7.23 (6.01–8.66)	1.02 (0.89–1.15)
Fourth-generation cephalosporins (J01DE)	po	1	–	–	–
	iv	83	5.0 (2.0–11.0)	8.24 (6.73–10.02)	1.19 (0.999–1.40)
Carbapenems (J01DH)	po	0	–	–	–
	iv	190	5.0 (2.0–9.0)	8.63 (7.37–10.08)	1.03 (0.92–1.14)
Combinations of sulfonamides and trimethoprim, incl. Derivatives (J01EE)	po	112	8.5 (4.0–21.0)	17.57 (13.98–21.94)	0.89 (0.77–1.01)
	iv	14	3.0 (1.8–4.0)	3.65 (2.54–5.15)	1.75 (1.12–2.48)
Other aminoglycosides (J01GB)	po	3	–	–	–
	iv	143	7.0 (3.0–11.0)	9.64 (8.35–11.09)	1.27 (1.12–1.43)
Fluoroquinolones (J01MA)	po	232	4.0 (2.0–8.0)	7.62 (6.52–8.89)	0.92 (0.83–1.01)
	iv	95	4.0 (2.0–7.0)	7.00 (5.72–8.53)	1.14 (0.97–1.33)
Glycopeptide antibacterials (J01XA)	po	10	7.0 (2.0–10.3)	13.15 (4.90–33.47)	0.79 (0.47–1.18)
	iv	353	5.0 (2.0–10.0)	8.69 (7.82–9.63)	1.10 (1.01–1.18)
Polymyxins (J01XB)	po	0	–	–	–
	iv	83	3.0 (1.0–6.0)	6.25 (5.07–7.64)	1.26 (1.05–1.49)
Other antibacterials (J01XX)	po	9	10.0 (5.5–12.0)	10.06 (6.65–14.84)	2.07 (1.13–3.27)
	iv	39	3.0 (1.0–7.0)	7.75 (4.92–11.98)	0.86 (0.66–1.09)
Antimycotics					
Antibiotics (J02AA)	po	3	–	–	–
	iv	154	5.0 (2.0–9.3)	9.00 (7.45–10.82)	0.94 (0.83–1.05)
Triazole derivatives (J02AC)	po	88	9.5 (3.0–23.0)	19.11 (14.82–24.44)	0.94 (0.79–1.11)
	iv	59	5.0 (2.0–18.0)	13.06 (9.27–18.17)	0.83 (0.68–1.01)
Other antimycotics for systemic use (J02AX)	po	2	–	–	–
	iv	82	5.0 (3.0–11.0)	10.84 (8.42–13.86)	0.96 (0.81–1.12)

^a^ Confidence Interval

## Discussion

AKI is a complication in clinical care that can be linked to a variety of anti-infectives. Signals indicating an association with AKI were detected in many categories of anti-infectives (Table 3). Polymyxins (ATC code: J01XB) had the highest crude ROR among 36 ATC-drug classes of anti-infectives (Table 3). The detailed mechanism of AKI by polymyxin remains unclear [24]. In our study, 106 out of 107 reports on polymyxin-related AKI indicated colistin (ATC code: J01XB01) administration, which was associated with an AKI incidence rate of approximately 10 to 55% [25]; the finding is consistent with as reported by Patek et al. that colistin had the highest AKI ROR in the FAERS database [7]. ROR signals were also associated with other anti-infectives. Aminoglycosides cause tubular cell toxicity, and vancomycin is linked to acute interstitial nephritis [25]. The incidence rate of nephrotoxicity is reportedly up to 58% in patients treated with aminoglycosides, but most recent reviews suggest rates of 5 to 15% [4]. All AKI reports in antimycotics were from amphotericin B. Amphotericin B causes AKI when used as monotherapy or combination therapy [4] and raises blood urea nitrogen (BUN) and serum creatinine in 80% of patients receiving a complete course of amphotericin B therapy [26].

The crude ROR values indicated the occurrence of AKI in anti-infectives-treated patients in the age ranges of 70–79 years, 80–89 years, and ≥ 90 years and in patients receiving anti-infective monotherapy or combination treatment (≥ 2 anti-infectives) (Table 5). However, the crude ROR is insufficient for assessing the relative strength of causality between drugs and AEs and only provides an approximation of the signal strength [14, 22].

The adjusted RORs after PS matching were used to make adjustments by multivariate logistic regression analyses, which mitigated the effects of covariates. The adjusted RORs tended to be higher in anti-infective combination therapy than in monotherapy. These results strongly suggested that the number of anti-infectives administered are related to the occurrence of AKI. A meta-analysis demonstrated that vancomycin plus piperacillin–tazobactam combination therapy had higher odds of AKI than vancomycin monotherapy, piperacillin–tazobactam monotherapy, and vancomycin plus cefepime or carbapenem combination therapy; the findings are suggestive of drug-drug interactions leading to AKI [7, 27]. We also observed higher AKI crude ROR of vancomycin plus piperacillin–tazobactam combination therapy than that of vancomycin monotherapy and piperacillin–tazobactam monotherapy. Since vancomycin plus piperacillin–tazobactam are common empiric antibiotic combinations, these findings have important implications in antimicrobial administration.

Aging is known to decrease renal drug elimination [28], which is associated with an increased risk of AKI by high drug exposure in the elderly. Rybak et al. reported AKI incidence rates of 5, 11, and 22% in patients treated with vancomycin monotherapy, aminoglycoside monotherapy, and combination therapy consisting of vancomycin and one aminoglycoside, respectively [29]. Thus, anti-infective combination therapy may increase the risk of AKI in older patients, which should be considered more carefully in clinical practice.

The time-to-onset analysis derived the daily numbers of onset events. We found that 48.1% of anti-infective-related AKI occurred within 5 days of treatment initiation, and the median for anti-infective-related AKI onset was 5.0 days post-initiation (Table 7, S1 Figure). We did not detect statistically significant differences of time-to-onset profiles among the different types of anti-infectives (14 ATC-drug classes) or the route of administration (iv versus po). The finding that there is no significant difference in AKI time-to-onset profile with respect to dosage forms (iv versus po) is interesting and warrants future studies for its validation.

There are inherent limitations in using SRS data. For example, the length of the post-launch period of the drug, the notification of AEs, missing data, over-reporting of AKI associated with antimicrobials, especially in the elderly, and under-reporting affect SRS analysis. There was no suitable comparison group, and data on patient characteristics were incomplete. The results of anti-infective combination therapy were partially refined using the PS matching technique. Therefore, adjusted RORs are likely to have improved odds accuracy compared to that of crude RORs.

It has been reported that angiotensin-converting enzyme inhibitors, angiotensin receptor blockers, nonsteroidal anti-inflammatory drugs, calcineurin inhibitor (cyclosporine, tacrolimus), sulfonamides, acyclovir, rifampin, phenytoin, interferon, and proton pump inhibitors are involved in AKI [5]. In this study, we did not evaluate the effect of concomitant drugs other than anti-infectives. More reliable epidemiological studies will be needed to derive the causal constraints from this analysis.

## Conclusions

The JADER database, which includes clinicians’ reports of potential AE concerns related to drugs, is a useful tool for pharmacovigilance because it is based on real-world data derived from clinical practice. We used adjusted RORs after PS matching to identify the risk of anti-infective-related AKI linked to the number of anti-infectives administered. We observed higher AKI crude ROR of vancomycin plus piperacillin–tazobactam combination therapy than that of vancomycin monotherapy and piperacillin–tazobactam monotherapy. The median period of anti-infective-related AKI onset was 5 days after therapy initiation. We believe that our data will provide guidance for reducing the incidence of AEs in elderly patients receiving polypharmacy.

## Supplementary Information

**Additional file 1: Figure S1.** Histograms and the corresponding Weibull shape parameters of AKIs associated with 14 anti-infective categories for which the number of cases was more than 100 and the lower limit of the 95% CI exceeded 1 in Table 3. Three different time-to-onset periods of reported cases per anti-infective category were the limit to calculate the Weibull shape parameter. Six anti-infective categories [fourth-generation cephalosporins (po), carbapenems (po), other aminoglycosides (po), polymyxins (po), antibiotics (po), other antimycotics for systemic use (po)] did not meet this limit.

**Additional file 2: Table S1.** Suspected drugs classified by the Anatomical Therapeutic Chemical classification system and the Defined Daily Dose (ATC/DDD).

## Data Availability

The datasets used and/or analyzed during the current study are available from the corresponding author on reasonable request.

## Abbreviations

AKI: Acute kidney injury
SRS: Spontaneous reporting systems
JADER: Japanese Adverse Drug Event Report
PMDA: Pharmaceuticals and Medical Devices Agency
MedDRA: Medical Dictionary for Regulatory Activities
WSP: Weibull shape parameter
