# Supplementary material for: Pharmacovigilance study of anti-infective-related acute kidney injury using the Japanese adverse drug event report database
Source: BMC Pharmacol Toxicol. 2021 Aug 30;22:47. doi: 10.1186/s40360-021-00513-x (PMC8404262; doi:10.1186/s40360-021-00513-x)
Supplement: Supplementary file 1 — Additional file 1: Figure S1. Histograms and the corresponding Weibull shape parameters of AKIs associated with 14 anti-infective categories for which the number of cases was more than 100 and the lower limit of the 95% CI exceeded 1 in Table 3. Three different time-to-onset periods of reported cases per anti-infective category were the limit to calculate the Weibull shape parameter. Six anti-infective categories [fourth-generation cephalosporins (po), carbapenems (po), other aminoglycosides (po), polymyxins (po), antibiotics (po), other antimycotics for systemic use (po)] did not meet this limit. [file 40360_2021_513_MOESM1_ESM.pptx]

## Slide 1
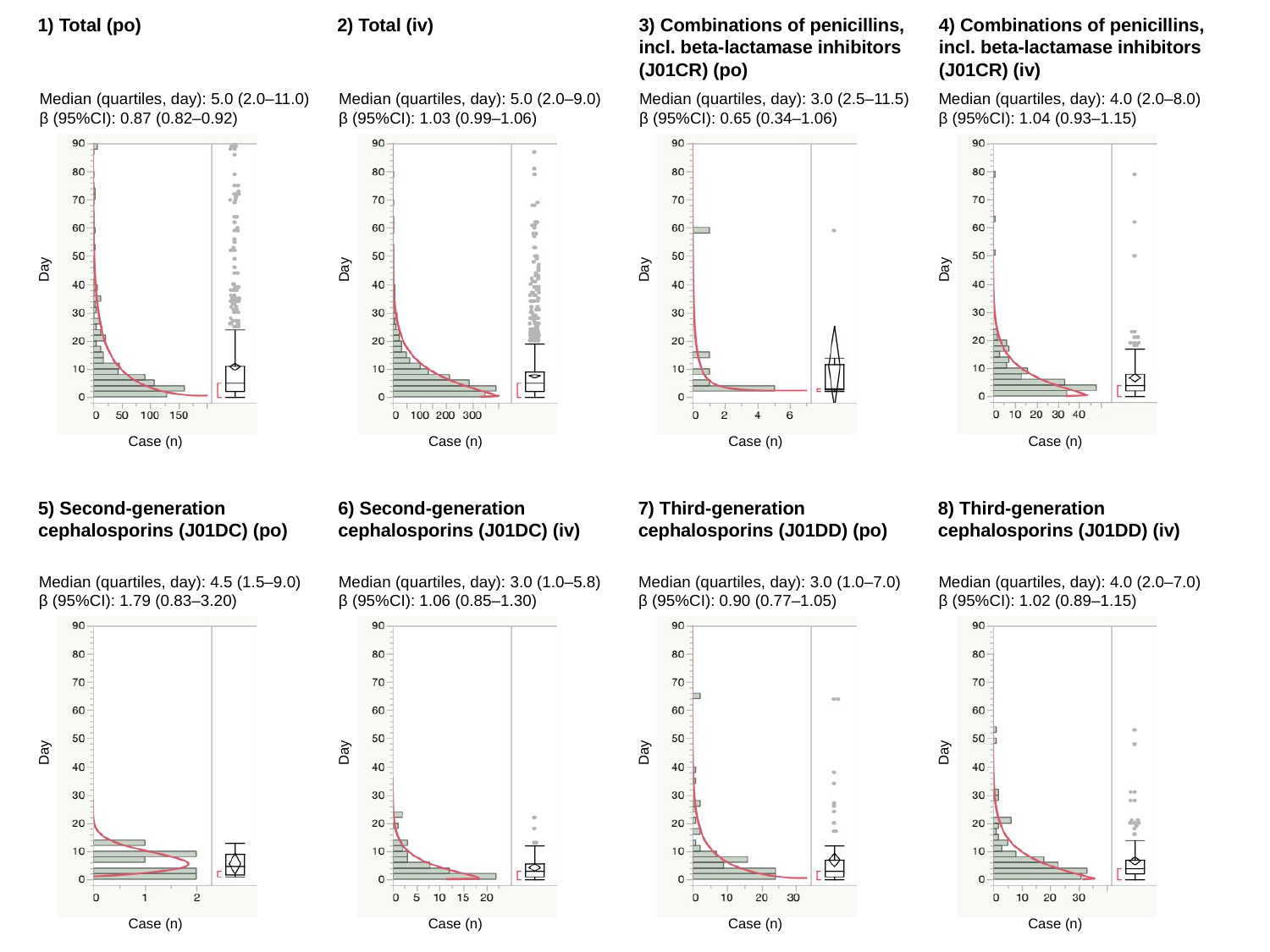

1) Total (po)
Median (quartiles, day): 5.0 (2.0–11.0)
β (95%CI): 0.87 (0.82–0.92)
Day
Case (n)
2) Total (iv)
Median (quartiles, day): 5.0 (2.0–9.0)
β (95%CI): 1.03 (0.99–1.06)
Day
Case (n)
3) Combinations of penicillins,
incl. beta-lactamase inhibitors
(J01CR) (po)
Median (quartiles, day): 3.0 (2.5–11.5)
β (95%CI): 0.65 (0.34–1.06)
Day
Case (n)
4) Combinations of penicillins,
incl. beta-lactamase inhibitors
(J01CR) (iv)
Median (quartiles, day): 4.0 (2.0–8.0)
β (95%CI): 1.04 (0.93–1.15)
Day
Case (n)
5) Second-generation
cephalosporins (J01DC) (po)
Median (quartiles, day): 4.5 (1.5–9.0)
β (95%CI): 1.79 (0.83–3.20)
Day
Case (n)
6) Second-generation
cephalosporins (J01DC) (iv)
Median (quartiles, day): 3.0 (1.0–5.8)
β (95%CI): 1.06 (0.85–1.30)
Day
Case (n)
7) Third-generation
cephalosporins (J01DD) (po)
Median (quartiles, day): 3.0 (1.0–7.0)
β (95%CI): 0.90 (0.77–1.05)
Day
Case (n)
8) Third-generation
cephalosporins (J01DD) (iv)
Median (quartiles, day): 4.0 (2.0–7.0)
β (95%CI): 1.02 (0.89–1.15)
Day
Case (n)

## Slide 2
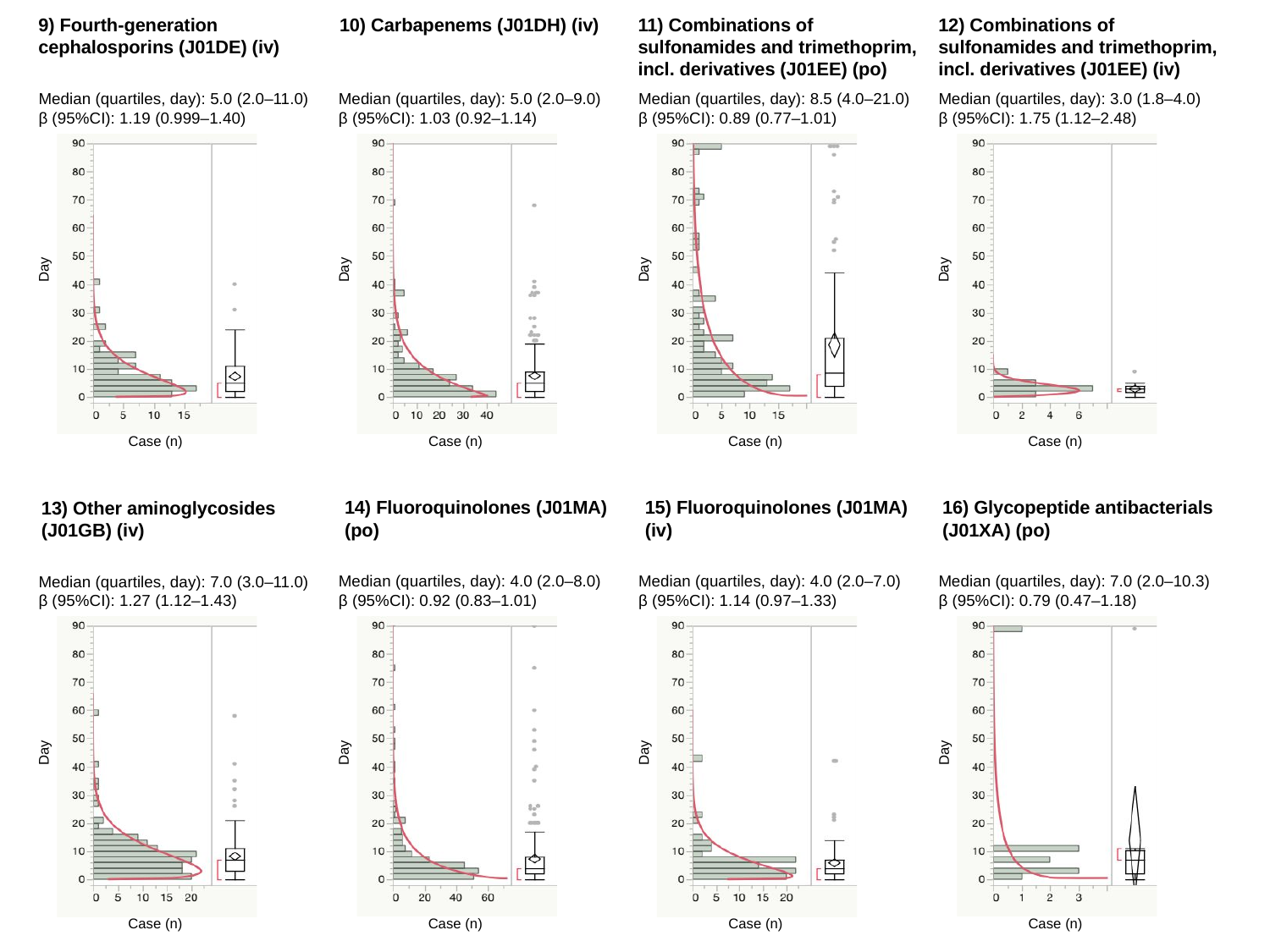

11) Combinations of sulfonamides and trimethoprim, incl. derivatives (J01EE) (po)
Median (quartiles, day): 8.5 (4.0–21.0)
β (95%CI): 0.89 (0.77–1.01)
Day
Case (n)
12) Combinations of
sulfonamides and trimethoprim,
incl. derivatives (J01EE) (iv)
Median (quartiles, day): 3.0 (1.8–4.0)
β (95%CI): 1.75 (1.12–2.48)
Day
Case (n)
9) Fourth-generation
cephalosporins (J01DE) (iv)
Median (quartiles, day): 5.0 (2.0–11.0)
β (95%CI): 1.19 (0.999–1.40)
Day
Case (n)
10) Carbapenems (J01DH) (iv)
Median (quartiles, day): 5.0 (2.0–9.0)
β (95%CI): 1.03 (0.92–1.14)
Day
Case (n)
14) Fluoroquinolones (J01MA)
(po)
Median (quartiles, day): 4.0 (2.0–8.0)
β (95%CI): 0.92 (0.83–1.01)
Day
Case (n)
15) Fluoroquinolones (J01MA)
(iv)
Median (quartiles, day): 4.0 (2.0–7.0)
β (95%CI): 1.14 (0.97–1.33)
Day
Case (n)
16) Glycopeptide antibacterials
(J01XA) (po)
Median (quartiles, day): 7.0 (2.0–10.3)
β (95%CI): 0.79 (0.47–1.18)
Day
Case (n)
13) Other aminoglycosides
(J01GB) (iv)
Median (quartiles, day): 7.0 (3.0–11.0)
β (95%CI): 1.27 (1.12–1.43)
Day
Case (n)

## Slide 3
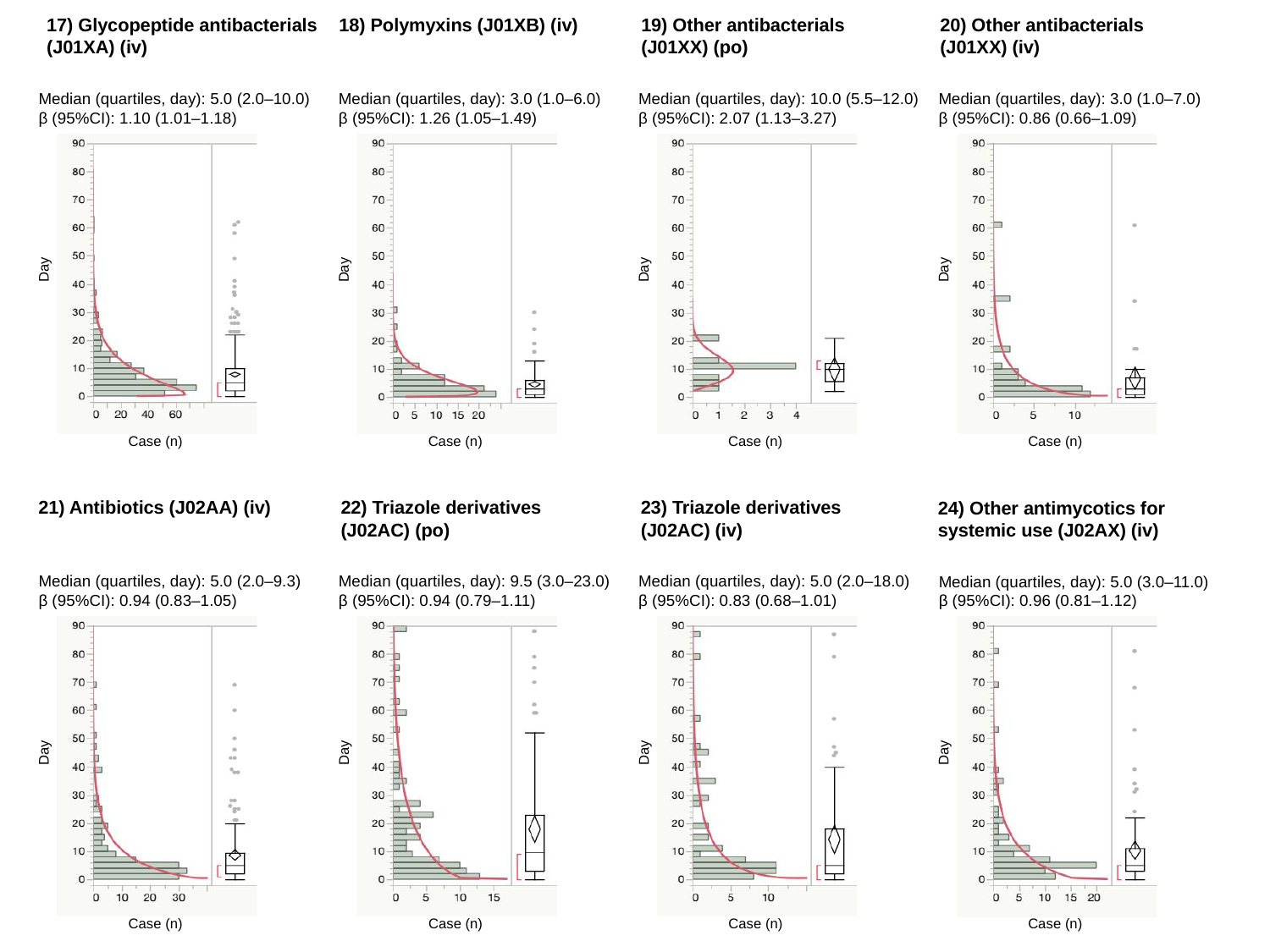

18) Polymyxins (J01XB) (iv)
Median (quartiles, day): 3.0 (1.0–6.0)
β (95%CI): 1.26 (1.05–1.49)
Day
Case (n)
19) Other antibacterials
(J01XX) (po)
Median (quartiles, day): 10.0 (5.5–12.0)
β (95%CI): 2.07 (1.13–3.27)
Day
Case (n)
20) Other antibacterials
(J01XX) (iv)
Median (quartiles, day): 3.0 (1.0–7.0)
β (95%CI): 0.86 (0.66–1.09)
Day
Case (n)
17) Glycopeptide antibacterials
(J01XA) (iv)
Median (quartiles, day): 5.0 (2.0–10.0)
β (95%CI): 1.10 (1.01–1.18)
Day
Case (n)
21) Antibiotics (J02AA) (iv)
Median (quartiles, day): 5.0 (2.0–9.3)
β (95%CI): 0.94 (0.83–1.05)
Day
Case (n)
22) Triazole derivatives
(J02AC) (po)
Median (quartiles, day): 9.5 (3.0–23.0)
β (95%CI): 0.94 (0.79–1.11)
Day
Case (n)
23) Triazole derivatives
(J02AC) (iv)
Median (quartiles, day): 5.0 (2.0–18.0)
β (95%CI): 0.83 (0.68–1.01)
Day
Case (n)
24) Other antimycotics for
systemic use (J02AX) (iv)
Median (quartiles, day): 5.0 (3.0–11.0)
β (95%CI): 0.96 (0.81–1.12)
Day
Case (n)
